# Selective bundle reconstruction for partial anterior cruciate ligament tears: High return to sport, excellent long‐term stability and function at 6‐year follow‐up

**DOI:** 10.1002/jeo2.70509

**Published:** 2025-11-03

**Authors:** Sergi Sastre, Diego Vasquez, Waldo González, Mariano Balaguer‐Castro, Dragos Popescu

**Affiliations:** ^1^ Knee and Arthroscopic Surgery Unit Hospital Clínic Barcelona Spain; ^2^ Hospital Regional Coyhaique Coyhaique Chile; ^3^ Department of Traumatology, Knee and Arthroscopy Unit, Clinica Alemana Universidad del Desarrollo Santiago Chile

**Keywords:** ACL augmentation, anterior cruciate ligament, knee stability, partial ACL tear, selective bundle reconstruction

## Abstract

**Purpose:**

Partial anterior cruciate ligament (ACL) tears account for 10%–27% of all ACL injuries. As an alternative to the well‐established option of complete reconstruction, selective bundle augmentation has recently been introduced into the repertoire of reconstruction techniques, aiming to preserve remnant tissue and provide superior biological and functional outcomes. The objective of our study is to assess long‐term clinical results, complication rates and return to sport after arthroscopic selective bundle reconstruction in patients with partial ACL tears.

**Methods:**

A retrospective cohort study was conducted. Patients with symptomatic partial ACL tears undergoing arthroscopic selective bundle reconstruction were included. Clinical stability and functional outcomes were assessed using anterior drawer, Lachman, pivot shift, Lysholm, Tegner scales, and a patient satisfaction score based on the Musculoskeletal Outcomes Data Evaluation and Management System scale. Statistical analyses were performed as described.

**Results:**

There was a statistically significant improvement in all stability tests after surgery (*p* < 0.001). The mean Lysholm score increased from 72.3 to 95.3, and the Tegner activity level from 2.05 to 4.86 (both *p* < 0.001). A high rate of return to sport was achieved with a low complication and re‐injury rate at long‐term follow‐up.

**Conclusions:**

Arthroscopic selective bundle reconstruction is a safe and effective treatment for partial ACL tears, providing excellent long‐term stability, functional recovery and patient satisfaction, with low complication and re‐injury rates. These findings support remnant‐preserving techniques in selected patients, though further prospective comparative studies are warranted.

**Level of Evidence:**

Level III.

**Abbreviations**
ACL: anterior cruciate ligament
AM: anteromedial
BMI: body mass index
BPTB: bone–patellar tendon–bone
MODEMS: Musculoskeletal Outcomes Data Evaluation and Management System
MRI: magnetic resonance imaging
PL: posterolateral
ROM: range of movement

## PURPOSE

Partial tears of the anterior cruciate ligament (ACL) are common injuries, accounting for approximately 10%–27% of all ACL tears [7, 30, 35, 36]. The primary mechanism of injury is sports activity, particularly in disciplines that involve pivoting and sudden directional changes, with or without direct contact. Depending on the initial position of the knee and the magnitude of the force involved, one or both bundles may be affected [10, 19].

Interest in modifying traditional complete reconstruction techniques in favour of selective reconstruction of injured structures in partial ACL tears, while preserving the remnant tissue, has grown based on an improved understanding of the ligament's biomechanics and biology. High‐quality residual fibres in an appropriate position may protect the graft during healing and ligamentization. Native blood vessels may enhance graft revascularization, and mechanoreceptors may contribute to preserving joint proprioception [5], potentially allowing for a shorter rehabilitation period and an earlier return to sport. Additionally, the preserved bundle can serve as a guide for proper tunnel placement [32].

**Figure 1:**
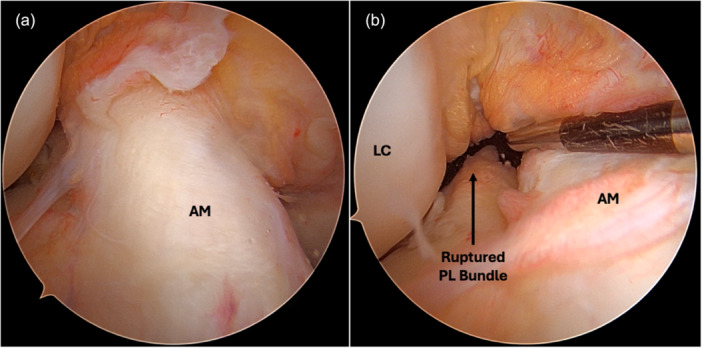
Intact anteromedial (AM) bundle (a). Tear of the posterolateral (PL) bundle (b). LC, lateral condyle.

The classical anatomy of the ACL describes two bundles: the anteromedial (AM) and the posterolateral (PL) [10, 15, 23, 28]. The definition of a partial ACL tear remains controversial. While the presence of continuous remnant fibres from the native femoral footprint to the native tibial footprint is a straightforward concept, the most widely accepted definition is based on the extent of fibre involvement. Specifically, a partial tear is defined as one in which less than 50% of the ligament fibres are torn [16, 40]. Magnetic resonance imaging (MRI) can aid in assessing these injuries but may sometimes only indicate, rather than confirm, a partial tear [11, 37]. Arthroscopy remains the gold standard for evaluating ACL injuries, as it allows accurate identification of the type of partial tear. Colombet et al. described four categories of ACL remnants based on their arthroscopic appearance and mechanical properties upon probing: completely absent ACL (50%), preserved PL bundle (16%), scarring onto the posterior cruciate ligament (23%) and preserved AM bundle (11%) [9] (Figure 1).

In patients with low physical demands, partial ACL tears may be managed conservatively through a targeted rehabilitation programme and extended monitoring until return to regular activity. However, studies have shown a higher risk of progression to a complete ACL tear in young, active individuals under the age of 20 who participate in pivoting sports [14, 35]. If conservative treatment fails, manifested by persistent instability or progression to a complete tear, surgical intervention should be considered [17, 25]. Surgical treatment may involve reconstruction similar to that performed for complete tears.

The objective of our study is to evaluate the long‐term clinical outcomes, complication rates, and return‐to‐sport rates following arthroscopic selective bundle reconstruction in patients with partial ACL tears. We hypothesized that arthroscopic selective bundle reconstruction for symptomatic partial ACL tears would significantly improve knee stability and functional outcomes compared with preoperative baseline.

## METHODS

A retrospective review was conducted on a cohort of patients who underwent ACL surgery at our department between January 2010 and December 2020. Ethical approval was obtained from the Health and Disability Ethics Committee of our centre, ethics approval number 2013‐01. Informed consent was obtained from all patients for the use of their data. No funding was required for this study.

### Eligibility criteria

Patients were eligible if they had an arthroscopically confirmed partial ACL tear (AM or PL bundle) suitable for selective bundle reconstruction (augmentation) and underwent the index procedure between January 2010 and December 2020. A minimum clinical follow‐up of 12 months and availability of pre‐ and post‐operative stability tests and functional scores were required.

Patients were excluded if a complete ACL tear was identified at arthroscopy, if multiligament knee injury or concomitant meniscal or chondral lesions requiring repair were present, or if clinical data were incomplete or follow‐up was <12 months. For the primary comparative analyses, cases reconstructed with grafts other than hamstring tendons were excluded a priori to avoid heterogeneity in outcomes.

### Definition of partial ACL tear

A partial ACL tear was defined as <50% of ligament fibres torn, determined intraoperatively during diagnostic arthroscopy. MRI served as supportive information only; confirmation of the diagnosis and identification of bundle involvement (AM or PL) were performed arthroscopically. Remnant status was characterized according to its arthroscopic appearance and response to probing as described in prior classifications; cases with complete fibre discontinuity or a non‐viable remnant were considered complete tears and were excluded.

### Surgical technique

All surgeries were performed by experienced knee surgeons (10+ years of experience) under general anaesthesia with a thigh‐high tourniquet. All diagnoses were first suspected clinically and with MRI, diagnostic arthroscopy was conducted to evaluate the remnant fibres and confirm the diagnosis of a partial tear. During the same surgical session, standardized augmentation was performed using the selected graft and an inside‐out technique, followed by femoral fixation with cortical buttons and tibial fixation with interference screws. Graft passage was performed with careful remnant preservation, avoiding notch impingement. After cycling the knee through the full range of motion, final tensioning and fixation were completed, and intra‐articular stability was reassessed arthroscopically.

Patients typically stayed one night in the hospital and completed at least one physiotherapy session before discharge.

Clinical outcomes were assessed using the anterior drawer test, Lachman test and Pivot Shift test, both preoperatively and post‐operatively. Additionally, outcomes were evaluated using the Tegner activity scale, Lysholm score, the Musculoskeletal Outcomes Data Evaluation and Management System (MODEMS) and return to pre‐injury sports activity [2, 6, 12, 31]. Objective knee stability tests, Tegner scale, Lysholm scale and MODEMS were performed by trained knee surgeons with ≥10 years of experience, following a standardized examination protocol. To minimize measurement bias, the same examiner conducted pre‐ and post‐operative evaluations at each scheduled visit whenever feasible.

Preoperative knee MRI was obtained on 1.5–3.0 T with a dedicated knee coil, using three orthogonal planes with fluid‐sensitive (PD/T2 fat‐suppressed) sequences and coronal T1‐weighted images. Studies were interpreted by board‐certified musculoskeletal radiologists.

### Post‐operative rehabilitation protocol

The rehabilitation protocol focused on pain and oedema management, joint range of movement (ROM) and quadriceps activation in the early weeks. As rehabilitation progressed, functional, proprioceptive and core‐strengthening exercises were introduced according to the recovery phase [22, 38]. Impact and plyometric exercises began no earlier than the third or fourth month, depending on the patient's condition [7]. Post‐operative follow‐ups were conducted at 2, 4 and 6 weeks, and at 3, 6 and 12 months by the treating surgeon. After completing the rehabilitation protocol, patients underwent MRI to assess graft ligamentization.

### Statistical methods

Categorical variables were presented as absolute frequencies and percentages. For quantitative variables, normality of distribution was assessed using the Shapiro–Wilk test.

For ordinal clinical tests (anterior drawer, Lachman and pivot shift), the Wilcoxon signed‐rank test was used to compare preoperative and post‐operative results, given the non‐parametric nature of the data. Regarding the functional scales (Tegner and Lysholm), the paired Student's *t* test was applied, as the data followed a normal distribution. A significance level of *p* < 0.05 was considered statistically significant. Patient satisfaction was assessed using the MODEMS scale, with results reported as mean, standard deviation, median and 25th and 75th percentiles, considering its ordinal nature.

Records with missing data were excluded from the statistical analysis to avoid bias in the comparisons. Statistical analyses were expressed with a 95% confidence interval in STATA v.18.5.

## RESULTS

A total of 396 surgeries were performed, from which 47 patients with partial ACL tears confirmed via arthroscopy were selected. At follow‐up, 43 patients were available, giving a follow‐up rate of 91.5% at a mean follow‐up time of 5.94 ± 2.60 years (range, 1.58–12 years).

The mean age of the patients was 30.02 ± 9.75 years (range, 16–55 years). The sex distribution was 30 men (69.77%) and 13 women (30.23%), consistent with the majority of published studies (Table 1). A history of previous knee injuries was present in 6 patients (13.95%), 5 had meniscal tears and 1 had a patellar dislocation.

**Table 1:** Demographic and characteristics of the patients.

Variable	Value
Mean age (year) (SD)	30.02 (9.75)
Mean BMI (kg/m^2^) (SD)	24.9 (3.5)
Sex	
Male	30 (69.67%)
Female	13 (30.23%)
Previous knee injuries	6 (13.95%)
Sports‐related injuries (*n*, %)	41 (95.35%)
Skiing	21 (48.84%)
Football	13 (30.23%)
Other sports	7 (16.28%)
Non‐sports‐related injuries (*n*, %)	2 (4.65%)
Laterality	
Right knee injuries (*n*, %)	23 (53.49%)
Left knee injuries (*n*, %)	20 (46.51%)
Injuries in dominant leg (*n*, %)	28 (65.12%)
Injuries in non‐dominant leg (*n*, %)	15 (34.88%)
MRI partial ACL tear diagnosis (*n*, %)	20 (46.51%)
Mean time from injury to surgery (months) (SD)	8.49 (4.75)

Abbreviations: ACL, anterior cruciate ligament; BMI, body mass index; MRI, magnetic resonance imaging; SD, standard deviation.

The most common injury mechanism was sports‐related (95.35%), with skiing accounting for 21 injuries (48.84%), football (soccer) for 13 injuries (30.23%) and 7 injuries (16.28%) from other sports. Non‐sport‐related causes, such as work or traffic accidents, were identified in only 2 patients (4.65%).

Regarding laterality, 23 injuries involved the right knee (53.49%) and 20 the left (46.51%). In terms of limb dominance, 28 injuries (65.12%) occurred in the dominant leg and 15 (34.88%) in the non‐dominant leg. When combining these two variables, most injuries occurred in the right and dominant limb (22 patients, 51.16%) (Table 1).

The diagnosis was made clinically in all patients using the Anterior Drawer test, Lachman test and Pivot Shift test. All patients subsequently underwent MRI, which reported a partial ACL tear in only 20 cases (46.51%); the remainder were classified as complete tears.

The graft of choice was predominantly hamstring tendons (40 patients, 93.02%). In one case each (2.33%), bone–patellar tendon–bone (BPTB), quadriceps tendon and allograft BPTB were used. For the analysis, only patients who underwent ACL reconstruction with hamstring grafts were included, to avoid the risk of losing clarity in the results.

The average time from injury to surgery was 8.49 ± 9.75 months (range, 1–20 months). After diagnostic arthroscopy, augmentation was performed in all patients. Among the total, the torn bundle was found in the AM in 23 patients (57.50%) and in the PL in 17 patients (42.50%). None of the patients had meniscal or cartilage injuries that required repair or would alter the rehabilitation protocol (Table 2).

**Table 2:** Surgical characteristics.

Variable	Value
Torn bundle—AM (*n*, %)	23 (57.50%)
Torn bundle—PL (*n*, %)	17 (42.50%)
Meniscal/cartilage injuries requiring repair	0
Mean tibial tunnel diameter (mm)	7.81
Tibial tunnel range (mm)	6–10
Mean femoral tunnel diameter (mm)	7.51
Femoral tunnel range (mm)	6–9
Mean operative time (min) (SD)	81.09 (23.50)

Abbreviations: AM, anteromedial; PL, posterolateral; SD, standard deviation.

The average tibial tunnel diameter was 7.81 mm (range, 6–10 mm), and the femoral tunnel diameter averaged 7.51 mm (range, 6–9 mm). Fixation was performed with a cortical button on the femoral side and an interference screw on the tibial side. The mean operative time was 81.09 ± 23.50 min (range, 51–145 min) (Table 2).

All patients who underwent arthroscopic ACL augmentation followed the same standardized rehabilitation protocol and attended follow‐up visits every 3 months during the first year and annually thereafter.

Two patients (4.65%) developed surgical site infections that required irrigation and antibiotic treatment. Late complications were observed in four patients (9.3%): two developed cyclops lesions requiring a second arthroscopy at 14 and 22 months, respectively, and the other two reported donor site paraesthesia. Only one patient (2.33%) experienced a recurrence of the injury 80 months after surgery, which has not required revision surgery due to the absence of clinical instability.

Statistically significant improvements were observed in all clinical tests performed. These differences indicate a clear enhancement in knee stability following selective bundle reconstruction.

Regarding the functional scales, the Tegner scale showed an increase from 2.05 ± 1.16 preoperatively to 4.86 ± 1.80 post‐operatively (*p* < 0.001), reflecting a significant improvement in the level of physical activity. The Lysholm score increased from 72.3 ± 11.6 to 95.3 ± 6.5 (*p* < 0.001), indicating a substantial functional improvement following the intervention.

**Figure 2:**
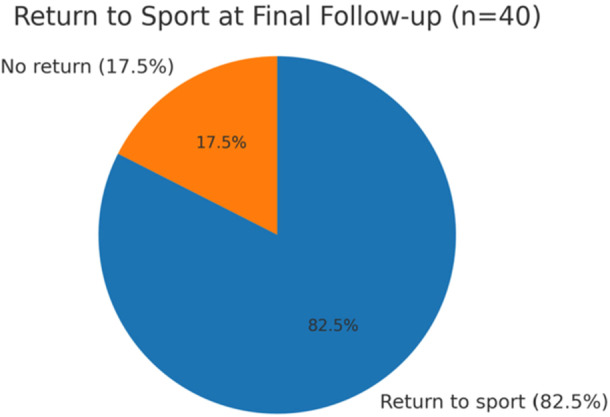
Return to sport at final follow‐up. Proportion of patients who resumed their pre‐injury sport after selective bundle reconstruction for partial ACL tears. ACL, anterior cruciate ligament.

Using the MODEMS satisfaction scale, 33 patients (82.5%) reported being very satisfied with the surgery, 5 patients (12.5%) were satisfied, 2 patients (5%) were neutral and no patients reported being dissatisfied or very dissatisfied, resulting in an average score of 4.78 (Table 3). Finally, all patients returned to sports at an average of 6.65 months post‐operatively; 33 patients (82.50%) returned to their previous level of activity and sport, 4 patients (10%) did not due to discomfort in the operated knee, and 3 patients (7.5%) were unable to return for other reasons, including other injuries, lack of time, work obligations and the pandemic (Figure 2).

**Table 3:** Satisfaction outcomes (MODEMS score).

MODEMS satisfaction score	*n*	%
5—Very satisfied	33	82.5
4—Satisfied	5	12.5
3—Neutral	2	5
2—Dissatisfied	0	0
1—Very dissatisfied	0	0
Total	40	100

Abbreviation: MODEMS, Musculoskeletal Outcomes Data Evaluation and Management System.

## DISCUSSION

This study aimed to evaluate the clinical outcomes of arthroscopic partial ACL augmentation for isolated bundle repair in cases of partial ACL rupture. These findings contribute to the growing body of evidence supporting the benefits of remnant‐preserving techniques in appropriately selected patients.

Partial ACL ruptures have traditionally presented significant challenges in both diagnosis and treatment due to variability in definitions and clinical presentation [20]. In this series, partial tears accounted for only 10.85% of all ACL surgeries, consistent with previous reports in the literature [5, 8, 18]. This relatively low incidence highlights the importance of maintaining a high index of suspicion and underscores the critical role of arthroscopy in establishing the diagnosis. Although partial tears were identified on MRI in only 20%–47% of patients [14], definitive diagnosis is ultimately achieved through arthroscopic evaluation, which allows dynamic assessment of the integrity and quality of the remaining bundle.

The principal finding of this study was a statistically and clinically significant improvement in knee stability following selective bundle reconstruction. All clinical stability tests—Anterior Drawer, Lachman and Pivot Shift—showed a highly significant reduction in anterior laxity post‐operatively (*p* < 0.001), indicating successful restoration of the joint's mechanical integrity [29]. These results are consistent with previous studies that have highlighted the benefits of preserving viable ligament fibres, including potential graft protection, enhanced revascularization and improved proprioceptive function [17, 24, 41]. Thus, by restoring both anteroposterior and rotational stability of the joint, the return to function was also successful.

A significant improvement in functional scores was observed, with the mean Lysholm score increasing from 72.3 to 95.3 (*p* < 0.001) between the preoperative and post‐operative assessments, and the Tegner activity level rising from 2.05 to 4.86 (*p* < 0.001). These results reflect a notable resumption of sports and physical activity, as 82.5% (*n* = 33) of patients were able to return to their pre‐injury level of sporting activity, with only one reinjury reported during long‐term follow‐up [1, 21]. Consistent with these outcomes, previous studies by Balemane et al. [3] and Sonnery‐Cottet et al. [34] have reported excellent joint stability, joint position sense and Lysholm scores at short‐term follow‐up after selective AM and PL bundle reconstructions. The present results align with these findings, demonstrating excellent functional scores, rotational and anterior stability of the knee, and a high rate of return to previous activity levels. Overall, the post‐operative outcomes are comparable to those reported after ACL double‐bundle reconstruction [4, 26].

The third key finding of this study was the high level of patient satisfaction. The mean MODEMS score was 4.78, with 82.5% of patients reporting being ‘very satisfied’ with the outcome of their surgery [13, 27, 33]. Satisfaction is an essential component of success, particularly in an active, often younger, population seeking return to sport [39].

In comparison with existing literature, these outcomes are consistent with those of other studies supporting the efficacy of selective reconstruction in partial ACL tears. The preservation of native fibres has been associated with improved proprioception, enhanced biological healing and reduced tunnel widening—all factors that likely contributed to the favourable results observed in this cohort.

The strengths of this study include a sample size larger than the average reported in the literature and an extended follow‐up period averaging nearly 6 years. This robust follow‐up allows for a meaningful evaluation of long‐term results, including graft performance, reinjury rates and functional maintenance over time. The follow‐up rate of 91.5% also enhances the reliability and generalizability of our findings. However, the study also has limitations, including its retrospective design, the absence of a control group—either patients with partial tears managed nonoperatively or those treated with complete ACL reconstructions—and the lack of scheduled post‐operative MRI follow‐up and objective assessments using arthrometric tools such as the KT‐1000. Despite these limitations, the favourable outcomes compare well with those reported in the literature and support the use of selective bundle reconstruction in this specific, carefully selected patient population.

The results of this study provide compelling evidence in support of arthroscopic selective bundle reconstruction in patients with partial ACL tears. This technique, performed in a carefully selected patient population, resulted in excellent long‐term outcomes in terms of knee stability, function, return to sport and patient satisfaction, with a low rate of complications.

## CONCLUSION

Arthroscopic selective bundle reconstruction for partial ACL tears is a safe and effective treatment, achieving significant restoration of joint stability, substantial functional improvement, and high long‐term patient satisfaction. In our cohort, with a median follow‐up of nearly 6 years, most patients returned to their pre‐injury level of sports activity, with a low complication rate and only one reported re‐injury. These findings strengthen the body of evidence supporting remnant‐preserving ACL techniques in carefully selected patients, offering potential biomechanical and biological advantages that may enhance rehabilitation and graft longevity. While the retrospective nature of this study and the absence of a control group are limitations, the results provide valuable long‐term data. Larger, prospective comparative studies incorporating standardized imaging and biomechanical assessments are warranted to better define clinical indications and validate the superiority of this technique over conventional ACL reconstruction.

## AUTHOR CONTRIBUTIONS

All the authors contributed to the design, analyses and reporting for this manuscript. Both authors read and approved the final submitted manuscript.

## CONFLICT OF INTEREST STATEMENT

The authors declare no conflicts of interest.

## ETHICS STATEMENT

Ethical approval was obtained from the Ethics Committee of our centre. Informed consent was obtained from all patients for the use of their data. Informed consent was taken from all patients for the use of data.

## Data Availability

Data available on request from the authors.
